# Supplementary material for: Anti-M1R/B6R antibody characterization and bispecific design for enhanced orthopoxvirus protection
Source: EMBO Mol Med. 2025 Sep 8;17(10):2713–34. doi: 10.1038/s44321-025-00299-z (PMC12514038; doi:10.1038/s44321-025-00299-z)
Supplement: Supplementary file 1 — Appendix [file 44321_2025_299_MOESM1_ESM.pdf]

Appendix

Anti-M1R/B6R antibody characterization and bispecific design for enhanced orthopoxvirus protection

Runchu Zhao[1,2,11], Lili Wu[2,11], Yi Zhang[1,2,11], Jianrong Ma[3,11], Dezhi Liu[1,2], Yuxuan Zheng[4], Tianxiang Kong[2,5], Renyi Ma[2], Zhengrong Gao[6,7], Yan Chai[2], Yuanlang Liu[8], Yi Tian[8], Yunxiang Xia[8], Yongzhi Hou[3], Jiahan Lu[3], Zhe Cong[3], Baoying Huang[9], Wenjie Tan[9], Jing Xue[3,*], George F. Gao[2], Qihui Wang[1,2,10,*]

Table of contents:

Appendix Figure S1 ········ 1
Appendix Figure S2 ········ 2
Appendix Figure S3 ········ 3
Appendix Figure S4 ········ 4
Appendix Figure S5 ········ 5
Appendix Figure S6 ········ 6
Appendix Figure S7 ········ 7
Appendix Figure S8 ········ 8
Appendix Figure S9 ········ 9
Appendix Figure S10 ········ 10
Appendix Figure S11 ········ 11
Appendix Figure S12 ········ 12
Appendix Figure S13 ········ 13
Appendix Table S1 ········ 14
Appendix Table S2 ········ 17
Appendix Table S3 ········ 18
Appendix Table S4 ········ 19
Appendix Table S5 ········ 20

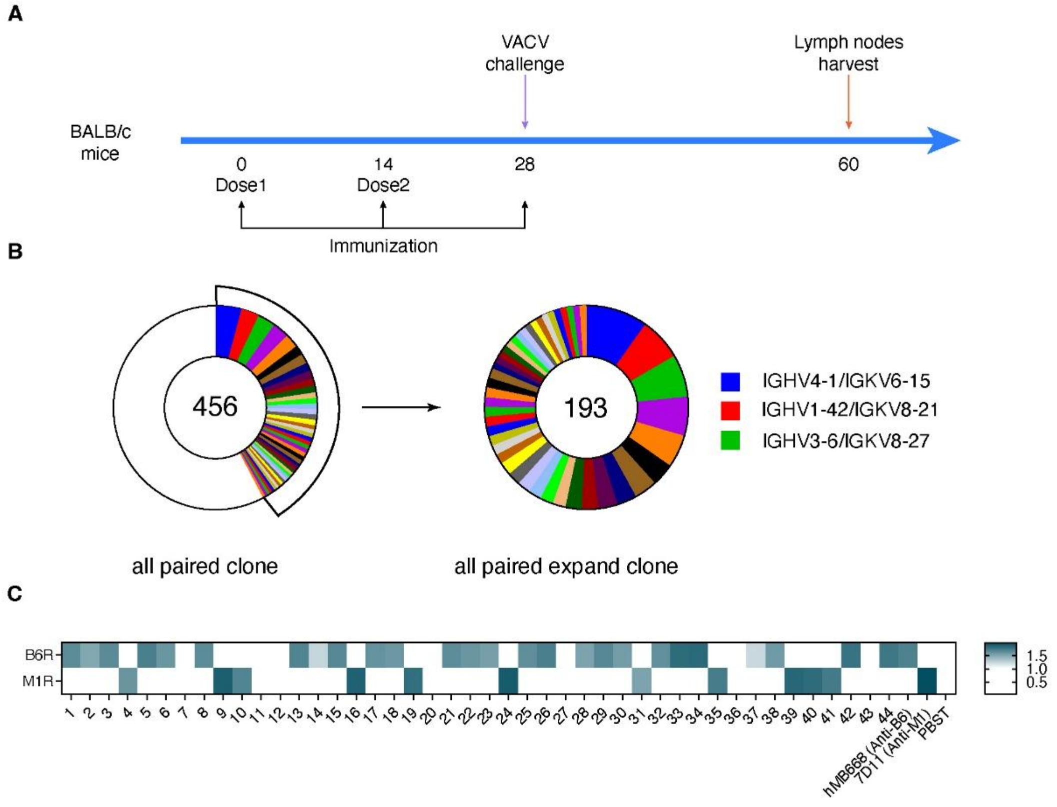

Appendix Figure S1. BCR repertoire elicited by MPXV M1R and B6R in mice.
(**A**) Schedule of immunization, viral challenge and sample collection. (**B**) Pie charts showing clonal expansion of MPXV-specific B cells in the germinal center. Colored slices represent expanded clones, with their size corresponding to the number of clones, while white slice indicates non-expanded clones. (**C**) Binding heatmap of 44 antibodies from expanded clones against B6R and M1R. Legend indicates the OD values tested by ELISA (*n* = 2).

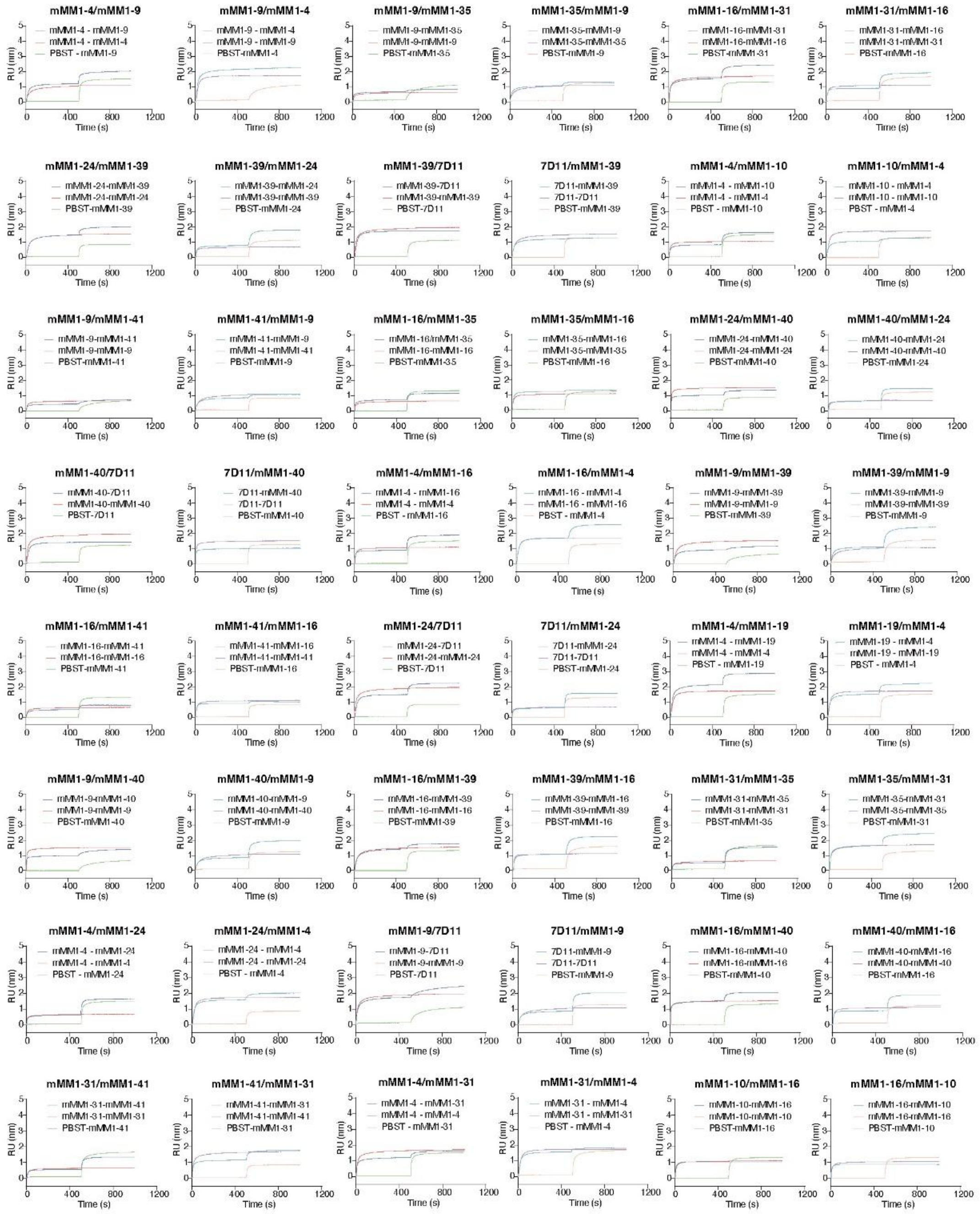

Appendix Figure S2. Pairwise competitive binding analysis of 11 anti-M1R monoclonal antibodies (MAbs) on MPXV M1R.

Octet RED96 was used to conduct pairwise competitive binding assays of 11 anti-M1R antibodies to classify them based on epitope specificity. 7D11 was used as an indicator.

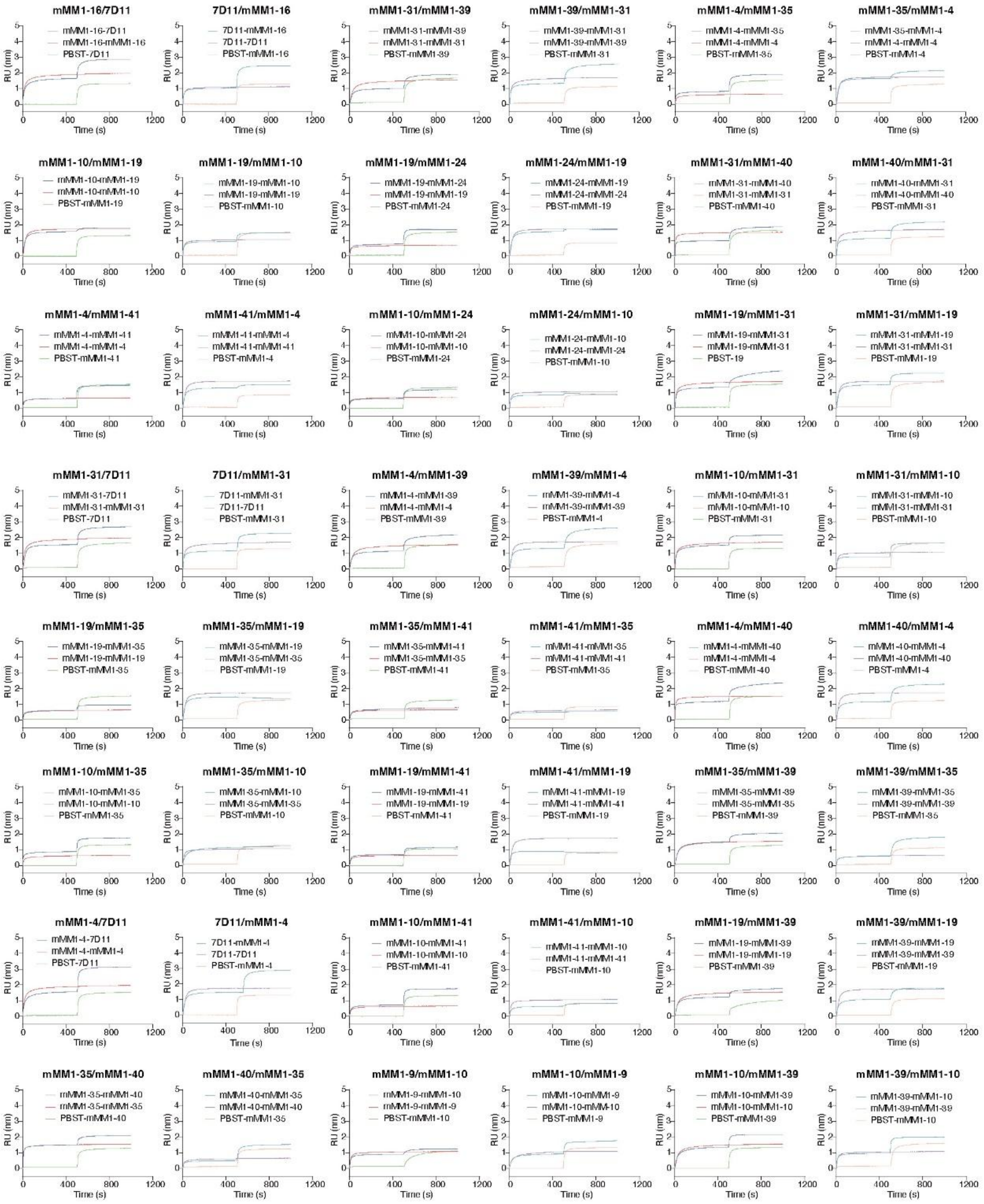

Appendix Figure S3. Pairwise competitive binding analysis of 11 anti-M1R MAbs on MPXV M1R, related to S2.

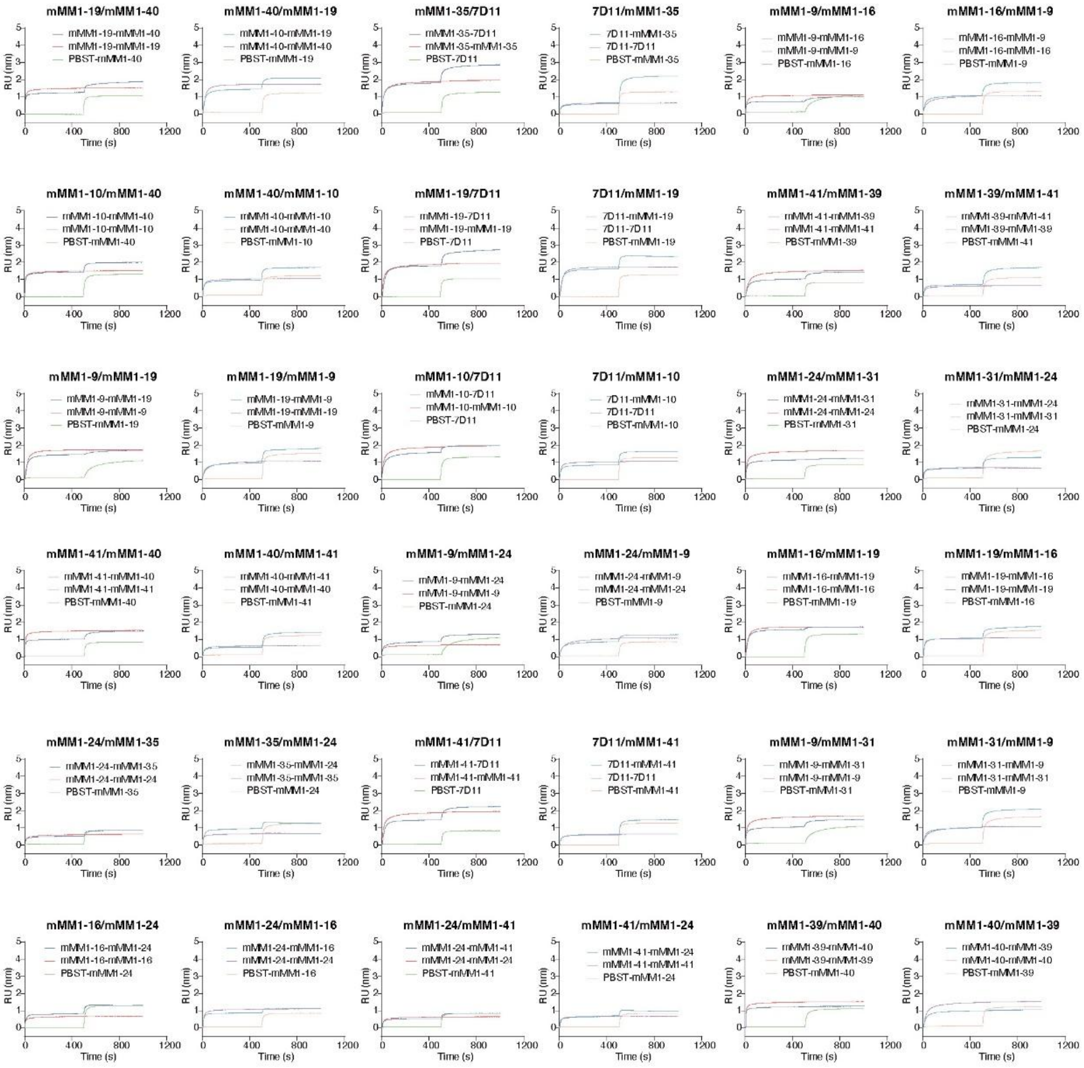

Appendix Figure S4. Pairwise competitive binding analysis of 11 anti-M1R MAbs on MPXV M1R, related to S2.

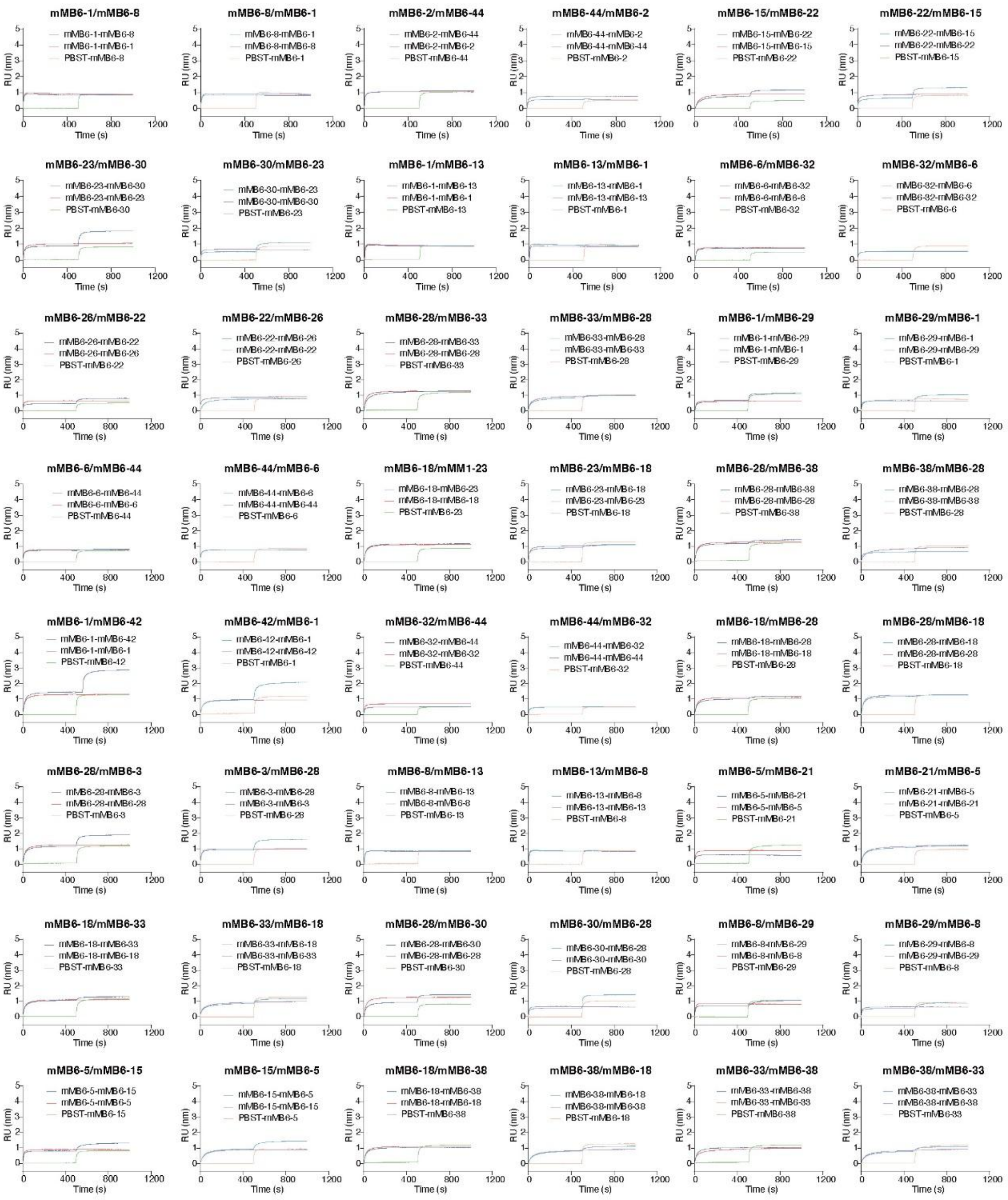

Appendix Figure S5. Pairwise competitive binding analysis within the four groups of anti-B6R MAbs on MPXV B6R.

Octet RED96 was used to conduct pairwise competitive binding assays of 21 anti-B6R antibodies to classify them based on epitope specificity.

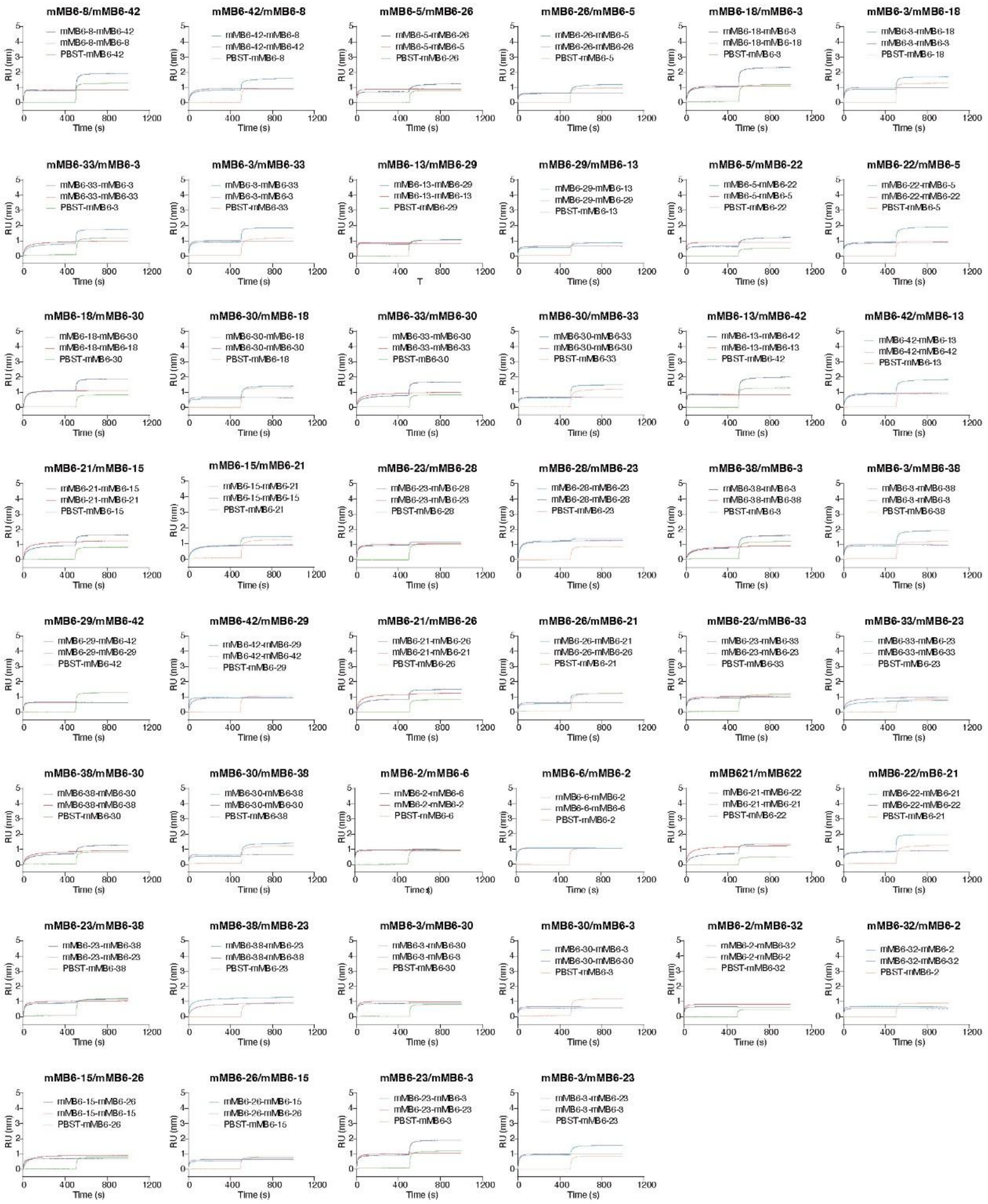

Appendix Figure S6. Pairwise competitive binding analysis within the four groups of anti-B6R MAbs on MPXV B6R, related to S5.

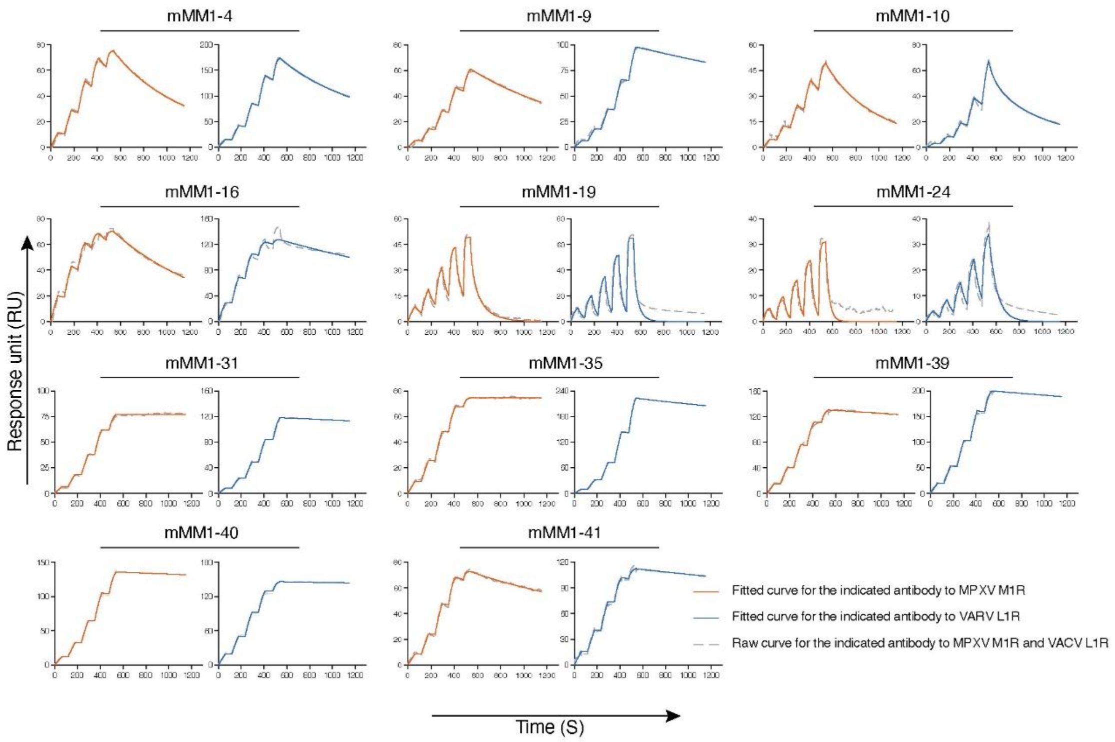

Appendix Figure S7. Binding characterization of 11 anti-M1R MAbs to MPXV M1R and its ortholog in VACV.

The indicated antibodies were captured by a Protein A chip, followed by serial dilution of MPXV M1R and VACV L1R flowing over the chip surface to assess the binding. Raw data are represented by dotted lines, while fitted curves for MPXV M1R and VACV L1R are shown as red and blue solid lines, respectively.

A

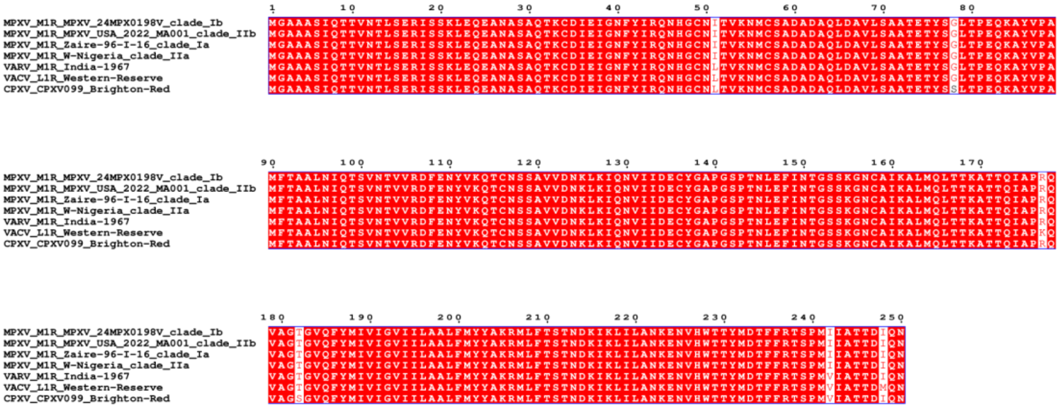

MPXV_M1R_MPXV_24MPX0198V_clade_Ib		100.00	100.00	100.00	99.20	98.40	98.40
MPXV_M1R_MPXV_USA_2022_MA001_clade_IIb	100.00		100.00	100.00	99.20	98.40	98.40
MPXV_M1R_Zaire-96-I-16_clade_Ia	100.00	100.00		100.00	99.20	98.40	98.40
MPXV_M1R_W-Nigeria_clade_IIa	100.00	100.00	100.00		99.20	98.40	98.40
VARV_M1R_India-1967	99.20	99.20	99.20	99.20		99.20	99.20
VACV_L1R_Western-Reserve	98.40	98.40	98.40	98.40	99.20		98.40
CPXV_CPXV099_Brighton-Red	98.40	98.40	98.40	98.40	99.20	98.40	

B

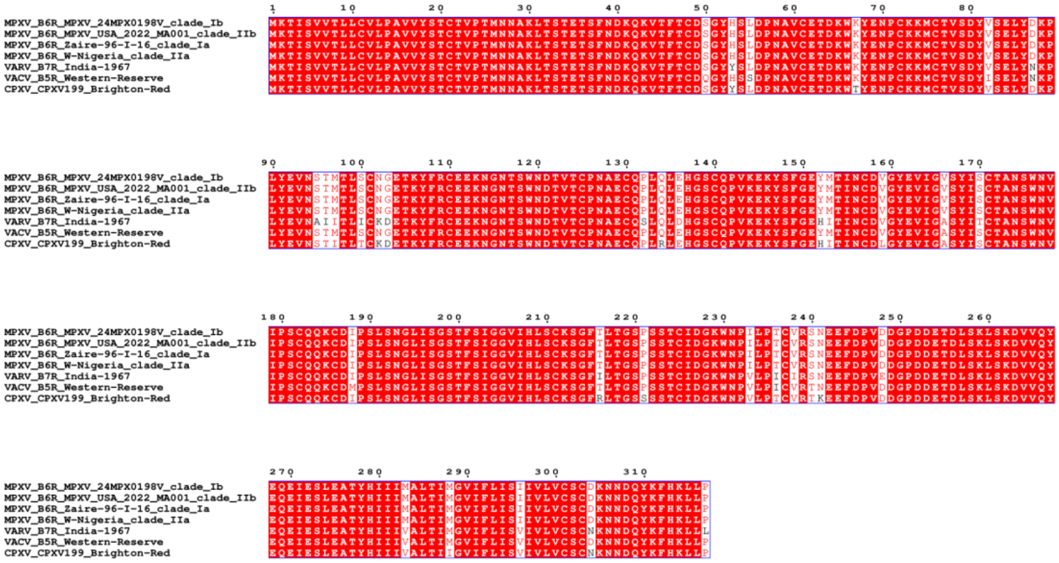

MPXV_B6R_MPXV_USA_2022_MA001_clade_Ib		100.00	100.00	100.00	92.74	96.53	93.69
MPXV_B6R_MPXV_24MPX0198V_clade_IIb	100.00		100.00	100.00	92.74	96.53	93.69
MPXV_B6R_Zaire-96-I-16 _clade_Ia	100.00	100.00		100.00	92.74	96.53	93.69
MPXV_B6R_W-Nigeria_clade_IIa	100.00	100.00	100.00		92.74	96.53	93.69
VARV_B7R_India-1967	92.74	92.74	92.74	92.74		93.06	94.01
VACV_B5R_Western-Reserve	96.53	96.53	96.53	96.53	93.06		93.38
CPXV_CPXV199_Brighton-Red	93.69	93.69	93.69	93.69	94.01	93.38	

Appendix Figure S8. Conservative analysis of M1R and B6R proteins among MPXV clade I, MPXV clade II, VARV, VACV and CPXV.

(**A-B**) M1R (**A**) and B6R (**B**) sequences were retrieved from publicly available reference genomes: PP601207.1 for the MPXV_24MPX0198V strain (clade Ib), ON563414.3 for the MPXV_USA_2022_MA001 (clade IIb), NC_003310.1 for the MPXV_Zaire-96-I-16 (clade Ia), KJ642615.1 for the MPXV_W-Nigeria (clade IIa), NC_001611.1 for the VARV_India-1967, NC_006998.1 for the VACV_Western-Reserve and NC_003663.2 for the CPXV_Brighton-Red.

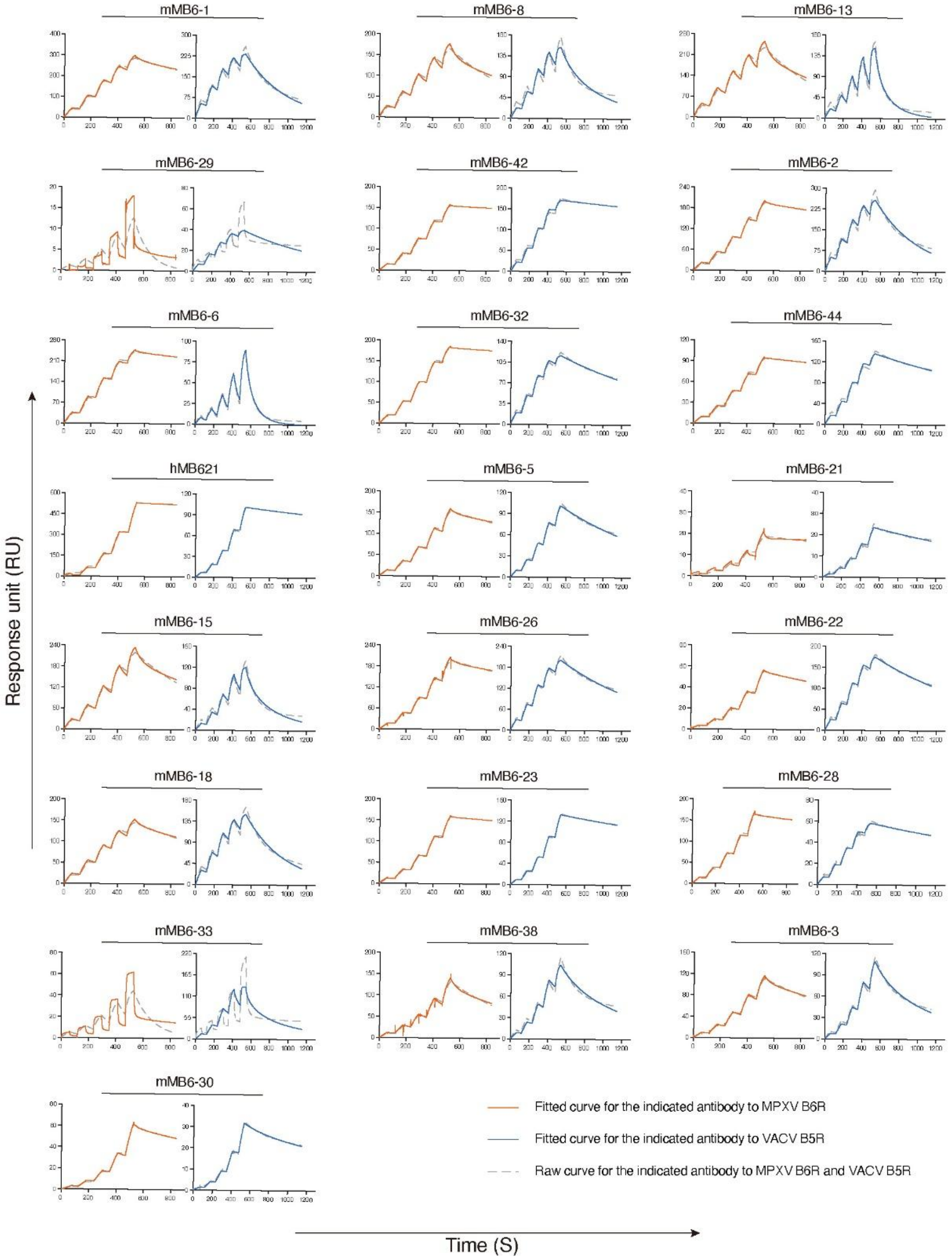

Appendix Figure S9. Binding characterization of 22 anti-B6R MAbs to MPXV B6R and its ortholog in VACV.

The indicated antibodies were captured by a Protein A chip, followed by serial dilution of MPXV B6R and VACV B5R flowing over the chip surface to assess the binding. Raw data are represented by dotted lines, while fitted curves for MPXV B6R and VACV B5R are shown as red and blue solid lines, respectively.

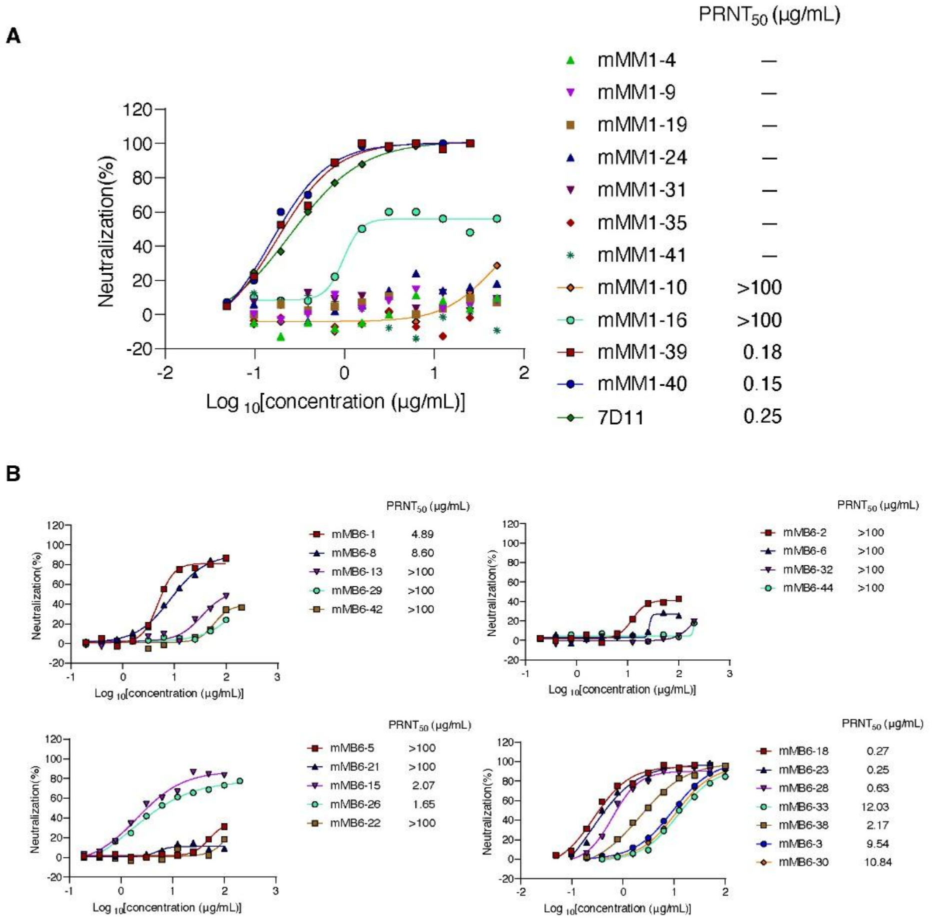

Appendix Figure S10. Neutralization curves of anti-M1R and anti-B6R MAbs against VACV.

Neutralization curves shown here are one representative data of two independent experiments (*n* = 2).

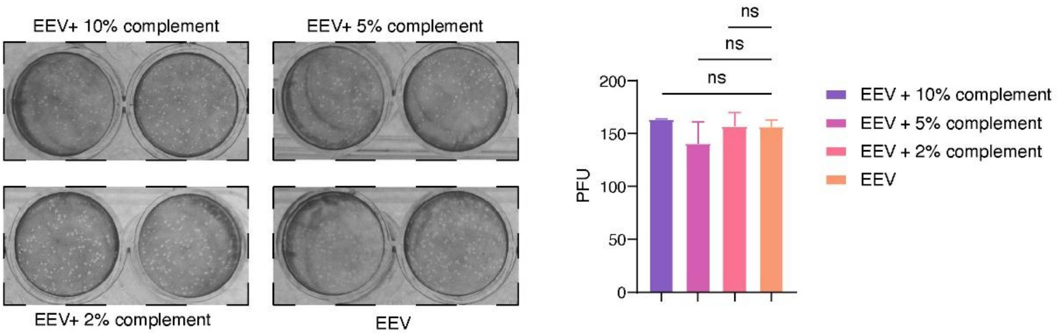

Appendix Figure S11. Evaluation of complement effects on VACV EEV infectivity. Effect of 10%, 5%, and 2% complement on VACV EEV plaque formation in Vero cells (n = 2). P = 0.8959 (EEV + 10% complement vs. EEV), P = 0.5083 (EEV + 5% complement vs. EEV), P > 0.9999 (EEV + 2% complement vs. EEV). Statistical analysis was performed by using ordinary one-way ANOVA with Dunnet's multiple comparisons test.

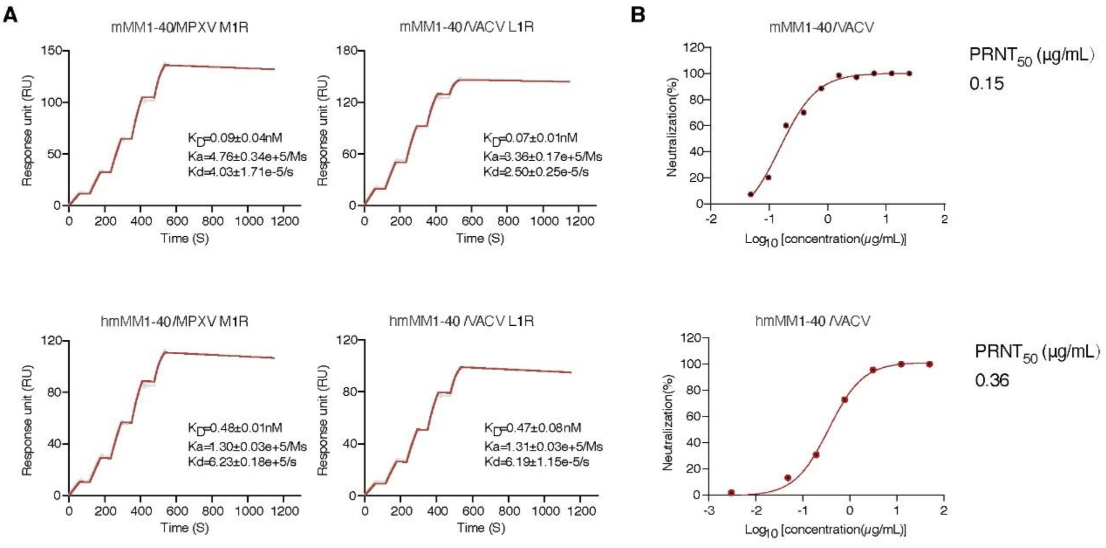

Appendix Figure S12. Comparative analysis of binding and neutralizing activities of mMM1-40 before and after humanization.

(**A**) Comparison of the binding affinities of mMM11-40 to MPXV M1R and VACV L1R before and after humanization. (**B**) Comparison of the neutralizing activities of mMM1-40 against VACV-IMV before and after humanization.

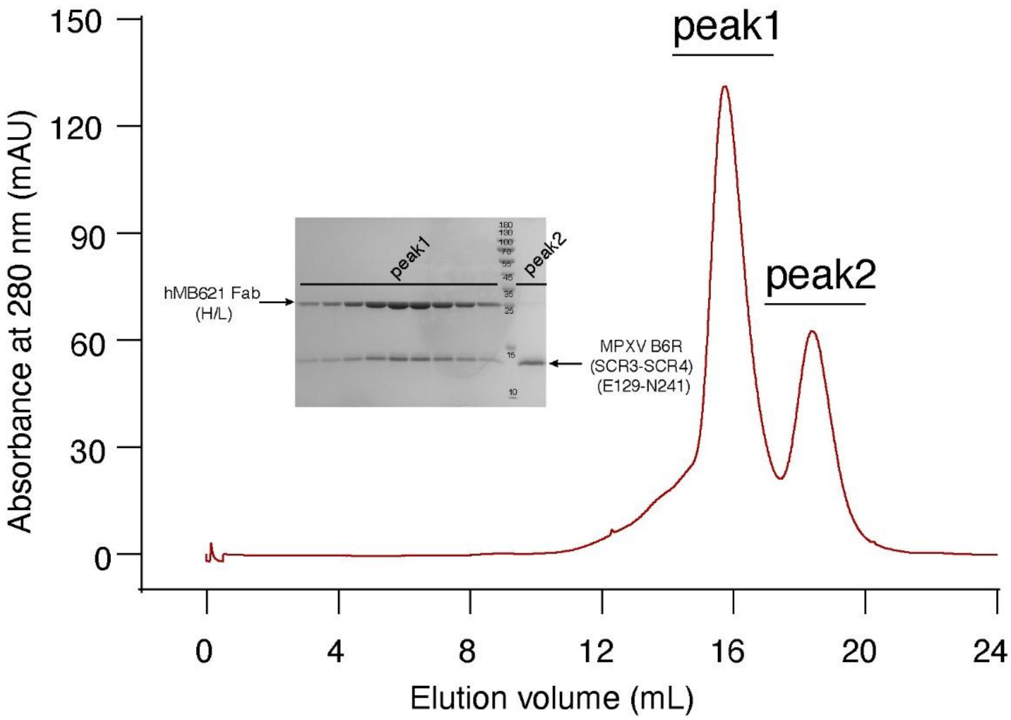

Appendix Figure S13. Gel-filtration analysis of hMB621 binding to B6R SCR3-SCR4. The reducing SDS-PAGE result showed peak 1 contains both hMB621 Fab and SCR3-SCR4 proteins, indicating these two proteins could form a complex in the gel-filtration assay.

Appendix Table S1. Germline analysis of MAbs

Antigen	Abs	Heavy chain variable gene sequence					Light chain variable gene sequence			
		V_H gene	V_H region nucleotide % homology to V_H gene	D_H gene	J_H gene	HCDR3 amino acids (aa)	V_L gene	V_L region nucleotide % homology to V_L gene	J_L gene	LCDR3 amino acids (aa)
M1R	mMM1-4	1-18*01	97.30%	N/A	3*01	AREGFAY	10-94*01	96.10%	1*01	QQYFKLPWT
	mMM1-9	9-2-1*01	96.60%	1-1*01	4*01	ARGPHYYGTNYDYTMDY	10-96*01 or 10-96*07	96.80%	1*01	QQSNTLPWT
	mMM1-19	1-9*02	95.60%	2-14*01	1*01 or 1*02	ARSAVVGTRFFDV	3-7*02	97.30%	4*01	QHSWEIPFT
	mMM1-24	2-6-7*01	97.60%	N/A	4*01	ARGRSYAMDS	12-44*01	96.80%	2*01	QQHYVTPYT
	mMM1-31	1-69-9*01	96.30%	2-14*01	4*01	TRGDYRDDFYAMDY	8-24*01	98.30%	5*01	QQHYSTPLT
	mMM1-35	5-6-5*01	92.00%	N/A	3*01	TNDGAWFAY	14-111*01	95.80%	4*01	LQYDEFPFT
	mMM1-41	2-9*02	95.50%	2-12*01 or 2-15*01 or 2-3*01	4*01	AVLLFLDSDVDY	12-44*01	95.40%	5*01	QHHYDPPLT
	mMM1-10	1-14*02	98.30%	2-2*01 or 2-7*01 or 2-9*01	4*01	ARDGYDGRGYAMDY	6-14*01	100%	6-14*01	LQHWNYPYT
	mMM1-16	1-87*02	96.20%	5-1-1*01 or 5-10*01 or 5-11*01	4*01	AKGDTYPAAMDY	12-41*01	97.50%	2*01	QHFWITPYT
	mMM1-39	1-7*02	99.00%	2-5*01 or 2-6*01	3*01	TRSDSTNYLFVY	8-21*01	98.70%	2*01	KQSYNLMYT
	mMM1-40	1-7*02	97.30%	2-5*01 or 2-6*01	3*01	ARSDYTNYVFEY	1-110*01	98.70%	2*01	SQSTHVPYT

	mMB6-1	4-1*02	97.30%	N/A	3*01	ARGSY	6-15*01	95.80%	2*01	QQYKSSPHT
	mMB6-8	1-47-7*01	95.60%	N/A	3*01	ARSTSWFTY	2-137*01	98.00%	2*01	MQHLEFPYT
	mMB6-13	4-1*02	98.60%	N/A	4*01	ARGAY	6-15*01	96.80%	2*01	QQYNNSPYT
	mMB6-29	14-3*02	93.90%	3-1*01 or 3-2*01 or 3-3*01	3*01	AGSGGTWGFAY	2-137*01	98.00%	2*01	MQHLERPYT
	mMB6-42	3-2*02 or 3-2-1*01	97.20%	3-1*01 or 3-2*01 or 3-3*01	3*01	AVGGSAWFAY	1-110*01	98.00%	1*01	SQSTRVPWT
	mMB6-2	1-18-4*01	94.20%	4-1*01 or 4-1*02	4*01	TRRTGTGAMDY	8-21*01	98.00%	1*01	KQSYNLPT
	mMB6-6	1-47-18*01	95.20%	1-1*01	3*01	TRSSLTTVVSPFAY	12-46*01	98.20%	2*01	QHFWGAPYT
B6R	mMB6-32	1-71-6*01	96.60%	1-1*02	2*01	ARSLLITVVAPFDY	12-46*01	96.10%	2*01	QPFWGPPYT
	mMB6-44	5-12*02	95.30%	2-14*01	3*01	SRQGYRYGGFAY	4-72*01	97.20%	4*01	QQWSSNPFT
	mMB6-5	4-1*02	98.00%	N/A	4*01	LRPADY	1-110*01	98.70%	1*01	SQSIHIPWT
	mMB6-21	4-1*02	98.00%	N/A	4*01	VRPMDY	1-110*01	98.70%	5*01	SQSTHVPLT
	mMB6-15	8-4-4*01	94.90%	1-1*01	3*01	ARVYYGSGYETY	12-41*01	98.20%	1*01	QHFWTNPW
	mMB6-26	8-4-4*01	96.30%	1-2*01	4*01	ARIVFTTAYAMDY	12-41*01	96.50%	1*01	QHFWTTPWT
	mMB6-22	5-9-4*01	97.60%	1-1*01 or 1-2*01	4*01	ARGPSYGGYAMDY	2-137*01	99.00%	2*01	MQHLEYPYT
	mMB6-18	1-37*01 or 1-37*02	96.20%	1-1*01	4*01	ANTVSSPWAMDY	15-103*01	96.50%	2*01	QQGQSFPYT
	mMB6-23	1-15*02	96.90%	N/A	2*01	TRGNHFDY	1-110*01	97.30%	2*01	SQSTHVPYT
	mMB6-28	14-3*02	96.30%	4-1*01	3*01	ARSPNWAWCAY	12-41*01	99.70%	1*01	QHFWSTPPT

	mMB6-33	1-20*02	97.60%	2-4*01 or 2-9*02	4*01	ASTVITPYAMDY	15-103*01	98.20%	2*01	QQGQSFPYT
	mMB6-38	1-42-1*01	96.60%	1-1*01	1*01 or 1*02	ARGEDYGRSPGYYFDV	4-68*01	99.00%	2*01	QQWSSNPPT
	mMB6-3	3-6*02	97.00%	2-2*01 or 2-7*01 or 2-9*01	3*01	ARDEGGYDQAWFPY	8-27*01	98.30%	2*01	HQFLSSST
	mMB6-30	14-1*02	97.30%	N/A	4*01	ARTGVMDY	8-27*01	99.00%	2*01	HQYLSSYT

Appendix Table S2. Statistics for data collection and refinement of MPXV M1R-hmMM1-40

	MPXV M1R-hmMM1-40
**Data collection**	
Wavelength (Å)	0.979183
Space group	$P2_1\ 2_1\ 2_1$
Cell Dimensions	
a, b, c (Å)	61.36, 72.35, 170.59
α, β, γ (°)	90.00, 90.00, 90.00
Resolution range (Å)	85.29-2.80 (2.95-2.80)
Unique reflections	18,631 (2,758)
Redundancy	7.3 (7.5)
Completeness (%)	96.0 (100.0)
I/σI	8.20 (1.50)
$CC_{1/2}$ (%)	98.6 (52.3)
**Refinement**	
R_{work}/R_{free}	0.2084/0.2960
Number of Atoms	
Protein	4,629
Ligand/ion	0
Water	0
B-factors	
Protein	61.0
Ligand/ion	—
Water	—
RMSDs	
Bond lengths (Å)	0.010
Bond angles (°)	1.160
Ramachandran Statistics (%)	
Favored	91.14
Allowed	8.53
Disallowed	0.33
Values in parentheses are for the highest-resolution shell	

Appendix Table S3. Contact between hmMM1-40 and MPXV M1R.

MPXV M1R	hmMM1-40		7D11	
	H chain	L chain	H chain	L chain
**E25**	R31(2, 1)		R31 (4, 1)	
**A26**	R31(5)		R31 (6)	
**N27**	R31(8), Y32(1), Y101(6)		R31 (2)	
**S29**	Y101(3)			
**Q31**	Y101(3, 1), T102(7), Y104(3)	Y37(2)	D102 (1), Y104 (2)	T34 (5), K36 (2)
**T32**	W33(1), Y101(6, 1), T102(15), Y104(1)		W33 (2), V101 (1), D102 (9), Y104 (2)	
**K33**	W33(2), Y50(5), E59(2), T102(4, 1), N103(6), Y104(16)	Y101(2)	W33 (3), D102 (3, 1), G103 (3), Y104 (22)	Y98 (1), L100 (1), W101 (4)
**D35**	W33(11, 2), Y50(6, 1),N52(4,1), Y57(24), N103(1)		W33 (11, 1), Y50 (6, 1), N52 (4, 1), T55 (1), Y57 (25, 1)	
**I36**			Y57 (1)	
**S58**	W33(5)		W33 (4)	
**A59**	T30(3), R31(3), Y32(1), W33(13), N52(10, 1), S54(3), T55(1)		T30 (3), R31 (2), W33 (14), N52 (10, 1), S54 (3), T55 (1)	
**D60**	T30(15, 1), R31(7), S54(9, 1)		T30 (16, 1), R31 (12), S54 (8, 1)	
**A61**	S54(5, 1), T55(1)		S54 (4, 1), T55 (4）	
**D62**	S54(4, 1)		S54 (3, 1), K74 (1)	
**A63**			R31 (3)	
**V122**	T55(1)		T55 (1)	
**K125**	T55(2, 1), G56(6), Y57(7)		G56 (3), Y57 (16), T58 (11)	
**K127**	Y57(11, 1), E59(5, 1)		Y57 (5), E59 (3)	
**S153**	T55(1)		T55 (1)	
Total	255, 16	4, 0	236, 11	13, 0

The numbers in parenthese of hmMM1-40 and 7D11 represents the numbers of van der Waals contacts contributed by the indicated residues in MPXV M1R/VACV L1R. The numbers behind comma suggest numbers of potential H-bonds between the pairs of residues. van der Waals contacts and H-bonds were analyzed at cutoff of 4.5Å and 3.5Å, respectively. M1R residues highlighted in bold blue on the left side of the table represent epitope sites shared by 7D11 and hmMM1-40.

Appendix Table S4. Statistics for data collection and refinement of MPXV M1R-mMM1-16

	MPXV M1R-mMM1-16
**Data collection**	
Wavelength (Å)	0.979183
Space group	$P4_3$
Cell Dimensions	
a, b, c (Å)	177.38, 177.38, 90.10
α, β, γ (°)	90.00, 90.00, 90.00
Resolution range (Å)	49.20-2.70 (2.86-2.70)
Unique reflections	150,678 (24,333)
Redundancy	5.3 (5.3)
Completeness (%)	99.9 (99.8)
I/σI	11.23 (1.78)
$CC_{1/2}$ (%)	99.6 (59.9)
**Refinement**	
R_{work}/R_{free}	0.2016/0.2742
Number of Atoms	
Protein	17,006
Ligand/ion	0
Water	0
B-factors	
Protein	60.0
Ligand/ion	—
Water	—
RMSDs	
Bond lengths (Å)	0.009
Bond angles (°)	1.131
Ramachandran Statistics (%)	
Favored	94.18
Allowed	5.82
Disallowed	0.00
Values in parentheses are for the highest-resolution shell	

Appendix Table S5. Contact between mMM1-16 and MPXV M1R.

MPXV M1R	mMM1-16	
	H chain	L chain
Q114		N28 (2), H30 (6, 1), W92 (7)
T115		W92 (1)
D117		I93 (4)
S118		W92 (11)
S119	Y102 (9), P103 (1)	F91 (2), W92 (4, 1), I93 (4), T94 (1), Y96 (5, 1)
A120	Y102 (17)	Y32 (3)
V122	R59 (1)	
D123	W33 (7, 1), R59 (11, 2), T101 (3), Y102 (5), P103 (2)	T94 (7, 1)
N124	T101 (13), Y102 (9)	
K125	W33 (4), Y52 (18), D55 (6, 1), D57 (6), T101 (4, 1)	
L126	T101 (2)	
Total	118, 5	57, 4

The numbers in parenthese of mMM1-16 represents the numbers of van der Waals contacts contributed by the indicated residues in MPXV M1R. The numbers behind comma suggest numbers of potential H-bonds between the pairs of residues. van der Waals contacts and H-bonds were analyzed at cutoff of 4.5Å and 3.5Å, respectively.
