# Supplementary material for: Anti-M1R/B6R antibody characterization and bispecific design for enhanced orthopoxvirus protection
Source: EMBO Mol Med. 2025 Sep 8;17(10):2713–34. doi: 10.1038/s44321-025-00299-z (PMC12514038; doi:10.1038/s44321-025-00299-z)
Supplement: Supplementary file 11 — Expanded View Figures [file 44321_2025_299_MOESM11_ESM.pdf]

Expanded View Figures

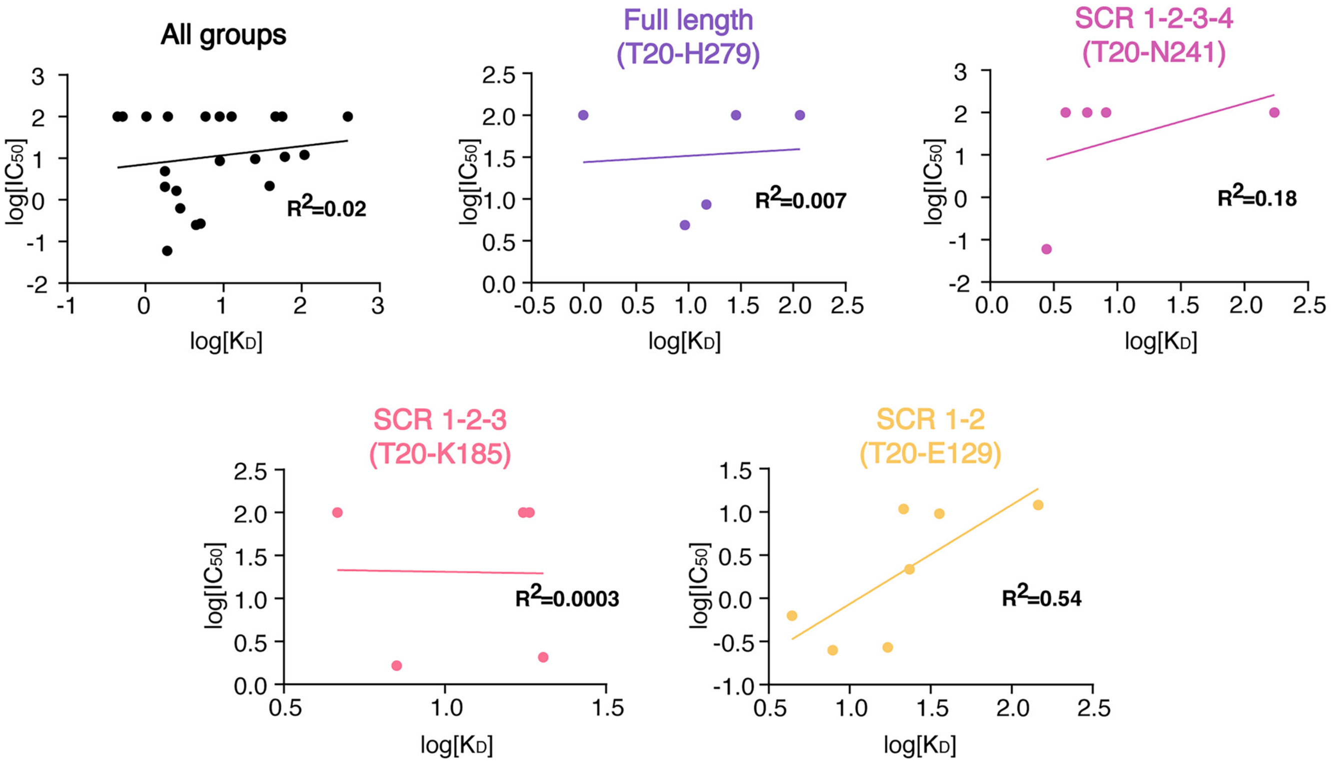

Figure EV1. Correlation between binding and neutralization of MPXV B6R-specific MAbs.

Log-transformed K_D (VACV B5R binding) and $PRNT_{50}$ (VACV neutralization) values of epitope-specific antibodies are plotted on the horizontal and vertical axes, respectively. The straight line represents a linear regression fit, with the corresponding R^2 value shown nearby.

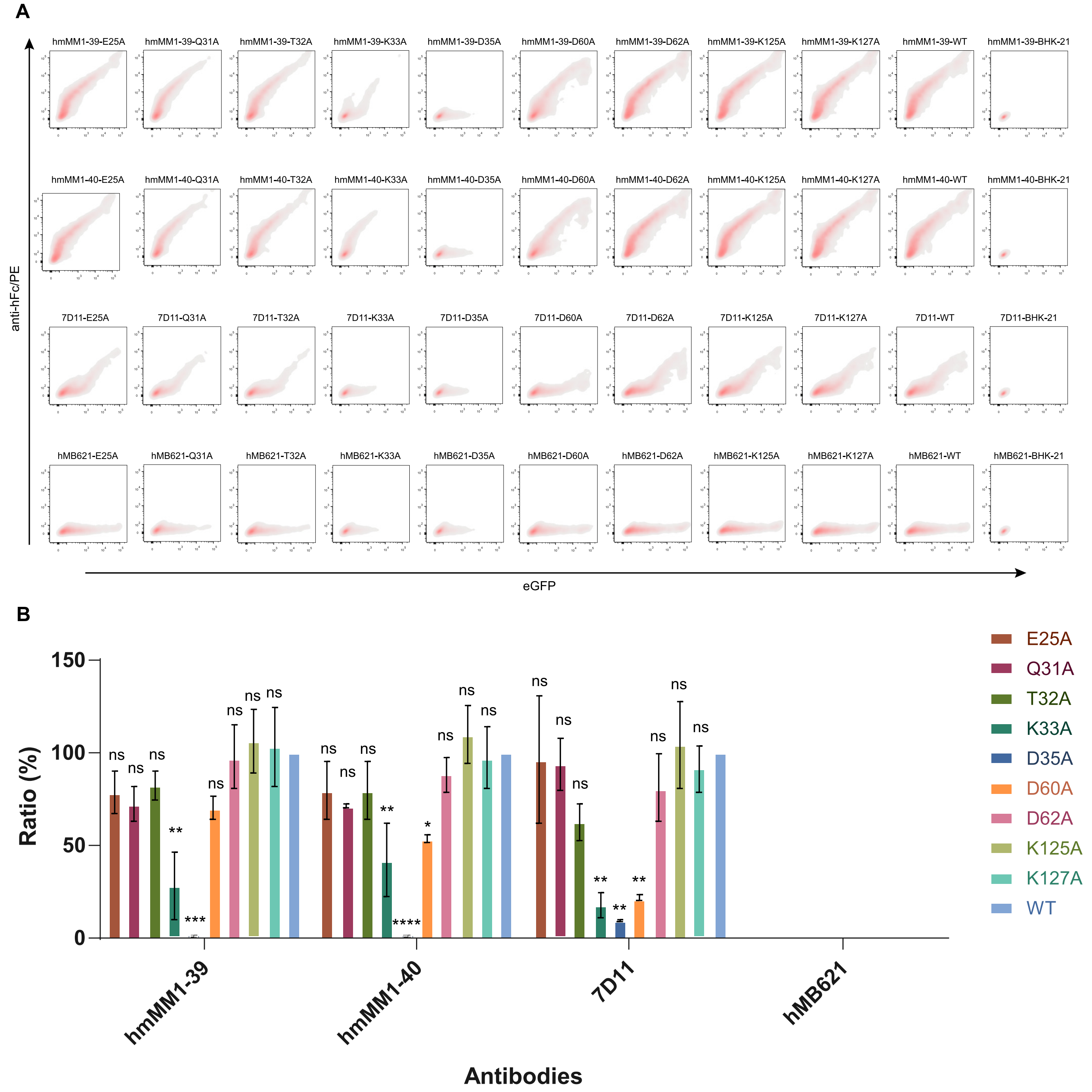

Figure EV2. Mutational analysis of key residues in MPXV M1R involved in interactions with both hmMM1-40 and 7D11.

(**A**) Density plot showing antibody binding to BHK-21 cells expressing wild-type or mutant M1R proteins. The x-axis (eGFP) indicates M1R protein expression, while the y-axis (PE) represents the level of antibody binding on the cell surface. (**B**) Mean fluorescence intensity (MFI) of PE within the eGFP-positive gated population, corresponding to antibody binding in cells expressing each M1R variant. In hmMM1-39 group, $P = 0.5066$ (E25A vs. WT), $P = 0.2574$ (Q31A vs. WT), $P = 0.6936$ (T32A vs. WT), $P = 0.0016$ (K33A vs. WT), $P = 0.0001$ (D35A vs. WT), $P = 0.2072$ (D60A vs. WT), $P = 0.9997$ (D62A vs. WT), $P = 0.9977$ (K125A vs. WT), $P = 0.9997$ (K127A vs. WT). In hmMM1-40 group, $P = 0.4651$ (E25A vs. WT), $P = 0.1839$ (Q31A vs. WT), $P = 0.4670$ (T32A vs. WT), $P = 0.0047$ (K33A vs. WT), $P < 0.0001$ (D35A vs. WT), $P = 0.0192$ (D60A vs. WT), $P = 0.9086$ (D62A vs. WT), $P = 0.9650$ (K125A vs. WT), $P = 0.9997$ (K127A vs. WT). In 7D11 group, $P = 0.9997$ (E25A vs. WT), $P = 0.9994$ (Q31A vs. WT), $P = 0.2051$ (T32A vs. WT), $P = 0.0028$ (K33A vs. WT), $P = 0.0014$ (D35A vs. WT), $P = 0.0039$ (D60A vs. WT), $P = 0.7775$ (D62A vs. WT), $P = 0.9997$ (K125A vs. WT), $P = 0.9941$ (K127A vs. WT). Each condition was tested in duplicate wells and the experiment was independently repeated twice. Data are mean ± SD ($n = 2$). Statistical analysis was performed by using ordinary one-way ANOVA with Dunnet's multiple comparisons test. *$P < 0.05$, **$P < 0.01$, ***$P < 0.001$, ****$P < 0.0001$, and ns $P > 0.05$. Source data are available online for this figure.

 © The Author(s)

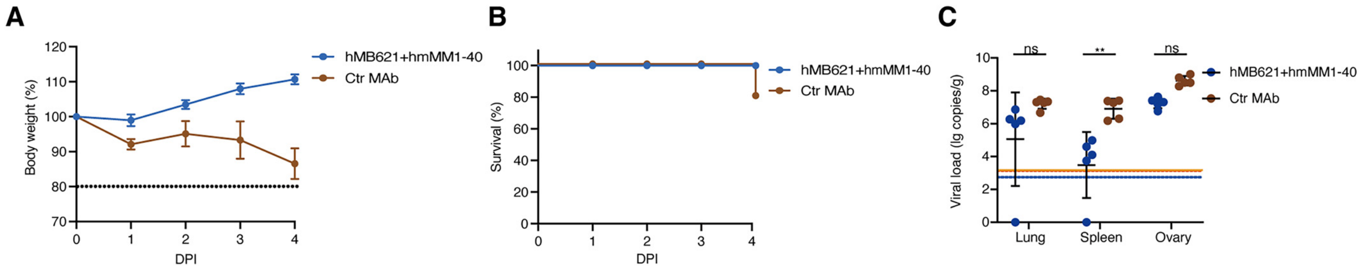

Figure EV3. In vivo protective efficacy of the hMB621 and hmMM1-40 antibody cocktail against MPXV infection.

(**A**, **B**) CB-17 SCID mice were i.p. injected with a 5 mg/kg cocktail of hMB621 and hmMM1-40, or an anti-SARS-CoV-2 RBD antibody as a control (Ctr MAb), at 4 h before and 4 h after challenge with a lethal dose of MPXV strain WIBP-MPXV-001. Body weight (**A**) and survival (**B**) were monitored daily until 4 dpi. Data are mean ± SD ($n = 4$ mice per group). (**C**) Viral titers in the lung ($P = 0.0834$ (hMB621+hmMM1-40 vs. Ctr MAb)), spleen ($P = 0.0033$ (hMB621+hmMM1-40 vs. Ctr MAb)) and ovary ($P = 0.3980$ (hMB621+hmMM1-40 vs. Ctr MAb)) were quantified using quantitative real-time PCR at 4 dpi. The red, yellow and blue dotted lines represent the LOD for the assays of the lung, spleen and ovary, respectively. Data are mean ± SD ($n = 4$ mice per group). Statistical analysis was conducted using two-way ANOVA with Dunnett's multiple comparisons tests. $^{**}P < 0.01$, and ns $P > 0.05$. Source data are available online for this figure.

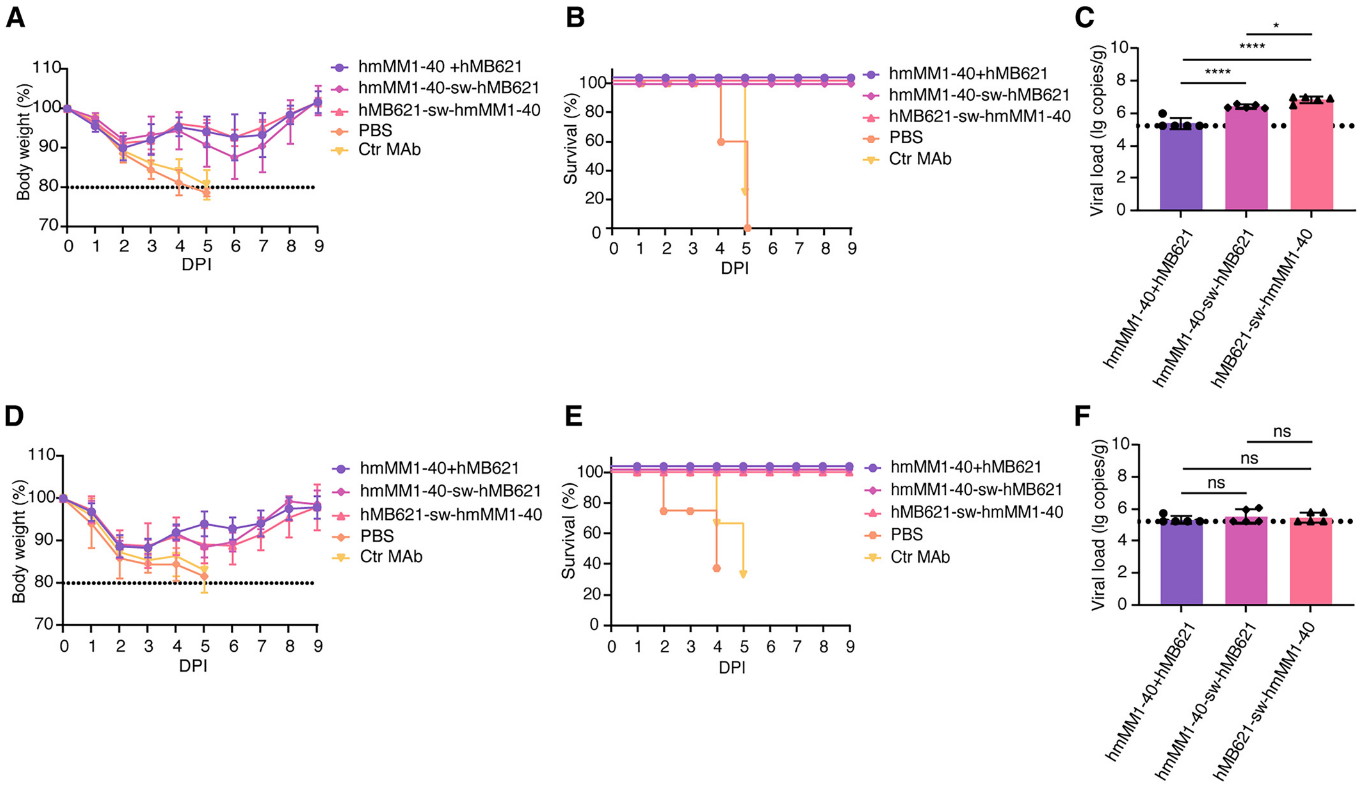

Figure EV4. Protective efficacy of antibody treatment at −24 h and +24 h against VACV.

(**A**, **B**, **D**, **E**). BALB/c mice ($n = 5$) received i.p. injections of 5 mg/kg of the indicated antibody or PBS either 24 h prior to (−24 h) or 24 h after (+24 h) i.n. challenge with a lethal dose of the VACV WR strain. Body weight (**A**, **D**) and survival (**B**, **E**) were subsequently monitored for 9 days. (**C**, **F**) Viral titers in the lungs at 9 dpi in mice that received antibodies 24 h before challenge, detected by quantitative real-time PCR, are shown in (**C**). $P < 0.0001$ (hmMM1-40 + hMB621 vs. hmMM1-40-sw-hMB621), $P < 0.0001$ (hmMM1-40 + hMB621 vs. hMB621-sw-hmMM1-40), $P = 0.0341$ (hmMM1-40-sw-hMB621 vs. hMB621-sw-hmMM1-40). Viral titers in the lungs at 9 dpi in mice that received antibodies 24 h after challenge, detected by quantitative real-time PCR, are shown in (**F**). $P = 0.5910$ (hmMM1-40 + hMB621 vs. hmMM1-40-sw-hMB621), $P = 0.8045$ (hmMM1-40 + hMB621 vs. hMB621-sw-hmMM1-40), $P = 0.9294$ (hmMM1-40-sw-hMB621 vs. hMB621-sw-hmMM1-40). Data are mean ± SD ($n = 5$ mice per group). Dotted line indicates LOD for the assay. Statistical analysis was performed by using ordinary one-way ANOVA with Dunnett's multiple comparisons test. *$P < 0.05$, ****$P < 0.0001$, and ns $P > 0.05$. Source data are available online for this figure.

 © The Author(s)
